# Miscellany

**Published:** 1906-05

**Authors:** 


### MISCELLANY.

THE BOECKEL SANITARIUM, at Gowanda, N. Y., is in complete working order in all its several departments. The buildings are new and are especially built for the treatment of alcoholism and other forms of narcomania. The system of ventilation is modern, the rooms are large and well furnished and there is an air of general comfort prevading the institution. The bath department includes Russian, Turkish, Saline, Electric, Needle, Shower, and Spray baths, which may be supplemented by massage and vibratory treatments. Physicians are invited to inspect the plant and its management.

---

THE STATE CIVIL SERVICE COMMISSION announces examinations to be held May 26, 1906, for the following positions: Assistant Civil Engineer, $5 to $6 a day; Bridge Designer, $1,500 to $1,800;

Civil Engineering Draughtsman, $4 to $5 a day; Junior Bridge Draughtsman, $900 to $1,200; Leveler, $4.50 to $5 a day; Electrical Engineer, Departments and Institutions, $900 and maintenance; Matron, Albany County Institutions, $300 and maintenance; Milk Expert, Department of Agriculture, $800 to $1,100; Page and Preparator in Entomology, State Museum, $480; Reformatory Guard, $480 to $720 and maintenance; Tracer, State Engineer's and State Architect's offices, $40 to $50 a month; Trained Nurse, State Institutions, $420 to $600 and maintenance.

The last day for filing applications for this examination is May 21st. Applications will be received until June 1st, for Assistants in Bacteriology, in Botany, in Entomology and in Horticulture at $720 a year in the Geneva Agricultural Experiment Station.

Application forms and detailed information may be obtained by addressing the Chief Examiner of the Commission at Albany.

